# Dynamic Effects in Nucleation of Receptor Clusters

**DOI:** 10.3390/e23101245

**Published:** 2021-09-24

**Authors:** Ivan V. Prikhodko, Georgy Th. Guria

**Affiliations:** 1 National Research Center for Hematology, 125167 Moscow, Russia; ivan.prikhodko@phystech.edu; 2 Moscow Institute of Physics and Technology, 141701 Dolgoprudny, Russia

**Keywords:** nucleation theory, receptor clusterization, von Neumann entropy, kinetic barrier, cell signaling

## Abstract

Nucleation theory has been widely applied for the interpretation of critical phenomena in nonequilibrium systems. Ligand-induced receptor clustering is a critical step of cellular activation. Receptor clusters on the cell surface are treated from the nucleation theory point of view. The authors propose that the redistribution of energy over the degrees of freedom is crucial for forming each new bond in the growing cluster. The expression for a kinetic barrier for new bond formation in a cluster was obtained. The shape of critical receptor clusters seems to be very important for the clustering on the cell surface. The von Neumann entropy of the graph of bonds is used to determine the influence of the cluster shape on the kinetic barrier. Numerical studies were carried out to assess the dependence of the barrier on the size of the cluster. The asymptotic expression, reflecting the conditions necessary for the formation of receptor clusters, was obtained. Several dynamic effects were found. A slight increase of the ligand mass has been shown to significantly accelerate the nucleation of receptor clusters. The possible meaning of the obtained results for medical applications is discussed.

## 1. Introduction

Modern concepts of critical phenomena in physicochemical nonequilibrium systems were formed after a well-known work, in which the concept of the critical size of a new phase nucleus was introduced [1]. For the first time, an expression for the nucleation rate was obtained in the work of Becker and Döring [2]. The approaches developed within the framework of this theory find their application in the interpretation of a wide range of phenomena in systems demonstrating first-order phase transitions [3,4,5,6]. Recently, approaches based on the concepts of nucleation theory were applied to analyze critical phenomena in biological systems [7,8,9].

In physical chemistry, the macroscopic features of nonequilibrium transitions were studied in detail [10,11,12,13,14]. It became clear that various biological systems (especially systems of the sensory type) undergo nonequilibrium transitions similar to a first-order phase transition [9,15,16].

The processes of cell receptor clustering play an important role in recognition and regulation [17,18,19]. The conditions for receptor clustering are actively being studied at present [20,21,22,23,24,25]. Several studies claim that clustering mechanisms are responsible for the sensitivity, specificity, and speed of ligand detection [26,27,28]. Significant progress has been achieved in the study of receptor clusters located on T cells [29], B cells [30], and platelets [31]. Nevertheless, relevant dynamic mechanisms of the formation of receptor clusters upon interaction with ligands remain poorly understood [27,28]: the conditions for critical nucleation during clustering are not clear. The influence of ligand mass on clustering processes is also unclear.

Clustering of receptors, under certain conditions, can occur on the cell surface even in the absence of any specific ligands [8,17,32]. These phenomena seem to be isomorphic to homogeneous nucleation in nonequilibrium systems [3,4,5].

The processes of receptor clustering, stimulated by external ligands, are similar to the processes of heterogeneous nucleation [9]. Investigating ligand properties that can significantly boost the probability of nucleation is a matter of great interest.

In this work, the goal is to apply the approaches developed earlier in the framework of nucleation theory and its physicochemical applications to the analysis of ligand-receptor biological clustering.

In the course of the analysis, an asymptotic expression reflecting the conditions necessary for the formation of receptor clusters was obtained. In particular, a slight increase of the ligand’s mass should significantly accelerate the nucleation of receptor clusters. The possible meaning of the obtained results for medical applications is discussed.

## 2. Materials and Methods

### 2.1. Background

The cell membrane is considered a two-dimensional surface that contains a certain number of receptors. Some of these receptors are in a free (not bonded) state. Other receptors, however, may have varying degrees of association with each other. With some probability, there can be associates consisting of two, three, or more receptors. Due to stochastic processes of association and dissociation, multimeric receptor clusters containing tens, and even hundreds, of receptors can arise [20,24,29,33,34].

In the present work, only clusters with a compact structure of sufficiently large sizes were assumed to be able to activate intracellular signaling pathways. Below, we will call such clusters productive.

Within the framework of the developed approach, nucleation processes are assumed to play an essential role in the formation of productive receptor clusters on the membrane. Clusters subcritical in size are believed to be statistically unstable: they spontaneously disintegrate with a high probability. At the same time, clusters of supercritical size are capable of explosive growth in systems with a sufficient level of receptor “supersaturation”. In those cases when the cell is in a metastable “waiting mode”, the concentration of single receptors $C_{r}$ on its surface is higher than the saturated concentration $C_{r0}$. However, clustering does not occur until nucleation seeds appear.

Elucidation of the conditions for forming supercritical receptor clusters on the membrane is of interest because the latter evolution ultimately leads to the formation of productive clusters capable of initiating intracellular signals. The spread of signals along the signaling pathways of the cell leads to a change in the gene expression of the cell nucleus that controls the response of the cell to external stimulation [35,36,37].

In this work, the receptor cluster is treated as a complex association of a group of receptors perceived as a separate long-lived structure. The structure of receptor clusters may differ significantly from droplets considered within the framework of traditional nucleation theory. The bonds of the receptors in the cluster may change, but on average bonds exist much longer than the sedentary life in a liquid phase. In the developed approach, oscillations of receptors have an essential role in the cluster dynamics.

According to the many-particle theories, each receptor cluster is a statistical object [38,39,40]. Dissipation issues in statistical ensembles have been studied previously in connection with the problem of energy equipartition over the degrees of freedom [41,42,43]. In particular, the energy equipartition over the degrees of freedom, even in simple finite-dimensional systems, was found to not always take place for times comparable to the characteristic oscillation time of individual cluster elements [44,45].

In cases when energy redistribution between the degrees of freedom is absent, and the oscillatory system is characterized by a high “quality factor”, formation of each new bond between the receptors may lead to energy consolidation after a period of whole cluster oscillations on the newly formed bond. Moreover, since the momenta of the particles in this state are opposed to the initial momenta, this process should break the bond. Thus, the retention of a new element by the cluster does not take place. These short-lived bonds in the cluster growth dynamics will not be considered.

The formation rate of the reaction complex is determined by the stochastic mixing of trajectories in the configuration space of the cluster. The energy redistribution over the degrees of freedom within the cluster itself affects the formation of the new long-lived bond to a much greater extent than the thermodynamic energy dissipation by the friction of cluster elements with the environment. Thus, the redistribution of energy over the degrees of freedom during receptor clustering should play a limiting role in nucleation processes.

### 2.2. Mathematical Model

In the theory of chemical reactions, the formation of long-lived bonds is described by the dynamics of reactants along a single reaction coordinate. The equation for assessing the reaction rate is:

(1) $$v=v_{0}\exp\left(-\Delta G^{\#}/k_{B}T\right)$$

where v is the reaction rate of bond formation, $v_{0}$ is the rate of the thresholdless reaction, $\Delta G^{\#}$ is the formation energy of the intermediate complex, $k_{B}$ is the Boltzmann constant, and T is the temperature [46,47].

In this case, “thermalization” (equipartition of energy) in the intermediate reaction complex proceeds along the transversal degrees of freedom to the reaction coordinate [48]. The problem of energy redistribution in critical states is common to many-particle theories [49,50,51]. This issue was studied by Izrailev and Chirikov [44]. An analysis of the one-dimensional dynamics of bound masses was carried out to study energy redistribution over the degrees of freedom. The authors considered the system of equations:

(2) $$m_{l}{\ddot{x}}_{l}=k\left(x_{l+1}+x_{l-1}-2x_{l}\right)\times \left\{1-\beta \left[{\left(x_{l+1}-x_{l}\right)}^{2}+{\left(x_{l}-x_{l-1}\right)}^{2}+\left(x_{l+1}-x_{l}\right)\left(x_{l}-x_{l-1}\right)\right]\right\}$$

where $m_{l}$ is the mass of the lth bound particle, $x_{l}$ is the deviation of the lth particle from the equilibrium position, k is the bond stiffness, and β is the nonlinear bond coefficient. The intermediate complex in this system corresponds to one of the unstable equilibrium positions.

Analysis of energy dissipation processes in receptor clusters having complex structures (characterized by Laplacian matrices [52,53]) implies the need to construct a multidimensional analog of Equation (2). In this respect, the movement of receptors included in the cluster was considered within the framework of the nonlinear matrix equation:

(3) $$M\ddot{X}=-kLX\left(I-\beta XL^{2}X\right)$$

where $X=diag\left(x_{1},y_{1},x_{2},y_{2}\ldots x_{N},y_{N}\right)$ is the matrix of receptor coordinates, $M=diag\left(M_{1},M_{1},M_{2},M_{2}\ldots M_{N},M_{N}\right)$ is the mass matrix, L is the Laplacian matrix for the graph of receptor bonds in the cluster, I is the identity matrix, k is the bond stiffness, and β is the nonlinear bond coefficient. The dimensions of the square matrices X, M, and L are given by the doubled number of receptors 2N×2N, where N is the number of receptors in the cluster.

Equation (3) was used in the present work to assess the efficiency of clustering processes in cases when receptor clusters can be considered as random graphs having limited degrees of the vertices [54].

To characterize the ability of the cluster configuration to redistribute energy over the degrees of freedom, the von Neumann entropy $H_{L}$ was used [55,56]:

(4) $$H_{L}=-\sum_{i}\frac{\lambda_{i}}{\sum_{j}\lambda_{j}}\ln\frac{\lambda_{i}}{\sum_{j}\lambda_{j}}$$

where $\lambda_{i}$ are eigenvalues of the Laplacian matrix L.

Expression (4) shows that the larger the von Neumann entropy is, the “denser” the spectrum of the eigenvalues of the Laplacian matrix. The eigenvalues are proportional to the square of the natural frequencies of the normal modes. The smaller the difference in natural frequencies at the corresponding degrees of freedom, the more efficient the redistribution of energy between the degrees of freedom [57]. In this regard, the von Neumann entropy appears to be a probabilistic measure of the energy redistribution over the degrees of freedom in a cluster with a given Laplacian matrix.

To assess the biochemical “potency” of a ligand, the association constant $K_{lr}$ is used:

(5) $$K_{lr}=C_{lr}/C_{l}C_{r}$$

where $C_{lr}$ is the concentration of receptor–ligand complexes, $C_{r}$ is the concentration of the receptors, and $C_{l}$ is the concentration of the ligands [58,59].

Typically, only a small proportion of receptors are associated with a ligand: $C_{lr}/C_{r}\ll 1$ [24]. Keeping this consideration in mind, Expression (5) for the “potency” of ligand $\varkappa_{l}$ can be easily transformed to the form:

(6) $$\varkappa_{l}\approx \frac{1}{m_{r}}m_{l}K_{lr}C_{l}$$

where $m_{r}$ is the receptor mass and $m_{l}$ is the ligand mass.

When the receptor can bind to several different ligands (for example, see [34,60,61]), the value characterizing the “potency” of the ligands ϰ was described by the following equation:

(7) $$\varkappa \approx \frac{1}{m_{r}}\sum_{i}m_{l}K_{lr}C_{l}$$

## 3. Results

### 3.1. The Potential Barrier of New Bond Formation in a Cluster

**Figure 1:**
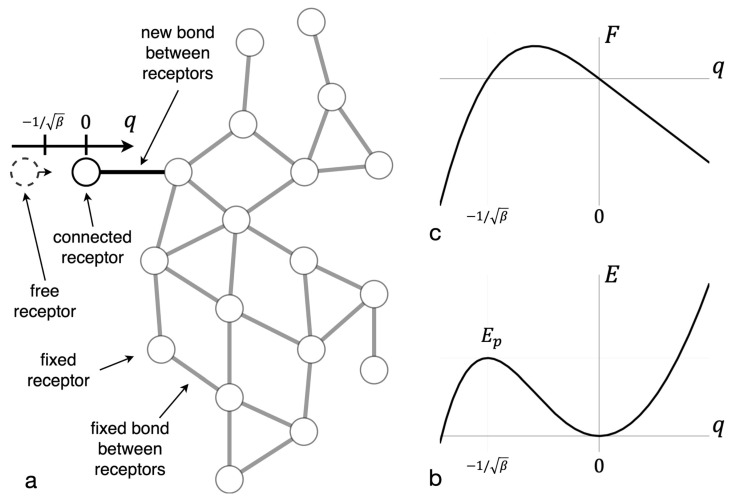
Force and energy dependence on the reaction coordinate. (**a**) The reaction coordinate *q* is shown for one bond in the cluster. (**b**) The dependence of bond force on that coordinate is shown. (**c**) The dependence of bond energy on the coordinate is shown. The extremum of the energy curve corresponds to an intermediate complex and has the energy of potential barrier $E_{p}$.

The receptor bond breaking energy was found in accordance with the Lagrange method of virtual displacements [62,63]. All receptor bonds except one were considered fixed. The latter elongates under the influence of a virtual external force from a stable equilibrium position to an unstable equilibrium position of an intermediate complex (see Figure 1a,b). The asymptotic expression for the height of the potential barrier, $E_{p}$, which must be overcome to break the bond, was found (see Figure 1c) and has the following form (see Appendix A for details):

(8) $$E_{p}=k/4\beta$$

where k is the bond stiffness and β is the nonlinear bond coefficient. Equation (8) reflects that in the ligand-receptor system, described by Equation (3), the bond force depends not only on the elongation of the emerging bond, but also on the adjacent bonds. It was taken into account that when one of the bonds is close to its critical length, its elongation is far bigger than the elongation of the adjacent bonds. Thus, the force on such bond can be assessed with high accuracy based only on the emerging bond elongation (see Appendix A).

**Figure 2:**
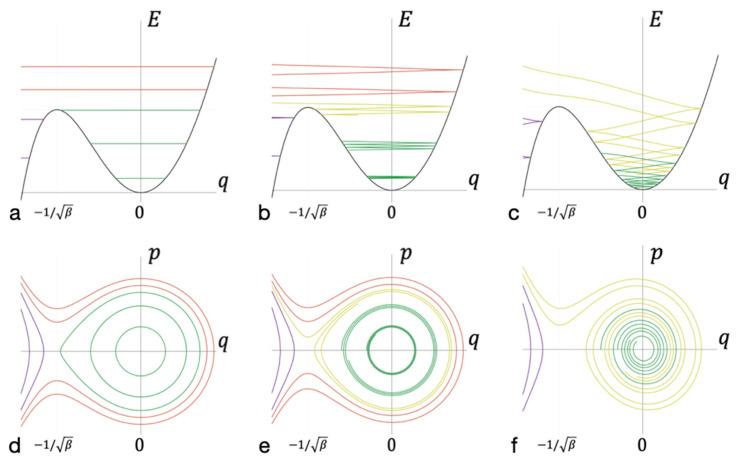
Energy and phase diagrams at different levels of dissipation. (**a**–**c**) The trajectories of the intermediate complex during the formation of a bond in the coordinate axes *q* of the oscillations of the entire cluster (abscissa) and the energy *E* at this coordinate (ordinate). (**d**,**e**) The same trajectories but in phase space in the coordinate *q* (abscissa) and momentum *p* (ordinate) of the oscillations of the entire cluster. Trajectories that do not overcome the potential barrier are marked in purple. Green—trajectories correspond to a bound state. Yellow—trajectories that lead to the formation of long-lived bonds. Red—trajectories that lead to the formation of short-lived bonds. Diagrams (**a**,**d**) correspond to motion without dissipation, (**b**,**e**) correspond to weak dissipation, and (**c**,**f**) correspond to strong dissipation.

In the absence of dissipation, the energy and phase diagrams look as shown in Figure 2a,d, respectively. A separatrix line separates the areas of stable bond and lack of bond; these areas are dynamically isolated. If the dissipation over the period of cluster oscillation is small with respect to oscillation energy, the phase and energy diagrams look as shown in Figure 2b,e. The smaller the value of dissipation during the period of cluster oscillation is, the smaller the width of the energy spectrum, which corresponds to the trajectories leading to the formation of long-lived bonds. In cases when the energy dissipation is high enough (see Figure 2c,f), a significant set of trajectories corresponding to $E>E_{p}$ lead to the formation of a long-lived bond.

### 3.2. The Kinetic Barrier of Bond Formation

To estimate the kinetic barrier $E_{k}$, the energy redistribution over the degrees of freedom of the cluster described by Laplacian matrix L was analyzed. The energy of the ligand-receptor system can transfer from the reaction coordinate to oscillations of other bonds in the cluster. Accordingly, the spectral decomposition of the matrix Equation (3) was carried out. Equations of motion were transformed to “normal coordinates”. The nonlinear terms in these equations act as perturbations of independent motion in the normal coordinates. Nonlinear perturbations lead to a slow (much longer than the mode oscillation period) energy redistribution between coordinates at low kinetic energy. However, when the kinetic energy exceeds the threshold calculated in the framework of the Kolmogorov–Arnold–Moser theory [57], a fast (four times shorter than the period of the mode oscillations) redistribution of energy between the modes should occur. That is, there must be an intracluster energy conversion. Concerning Equation (3), the value of the threshold, found within the framework of the Kolmogorov–Arnold–Moser theory, $E_{k}$, reflects the kinetic barrier of bond formation.

For the value of the kinetic barrier $E_{k}$, in this work, the following expression was obtained:

(9) $$E_{k}=\frac{3\pi }{1+\varkappa }\frac{e^{H_{L}}}{N}E_{p}$$

where $H_{L}$ is the von Neumann entropy of the Laplacian matrix of bonds in a cluster, N is the number of receptors in a cluster, and ϰ is the ratio of the total ligand mass to the total receptor mass in the cluster (see Appendix A for details). Thus, the kinetic barrier depends not only on the reaction coordinate dynamics, but also on the dynamics of all receptors in the cluster described by Laplacian matrix L.

**Figure 3:**
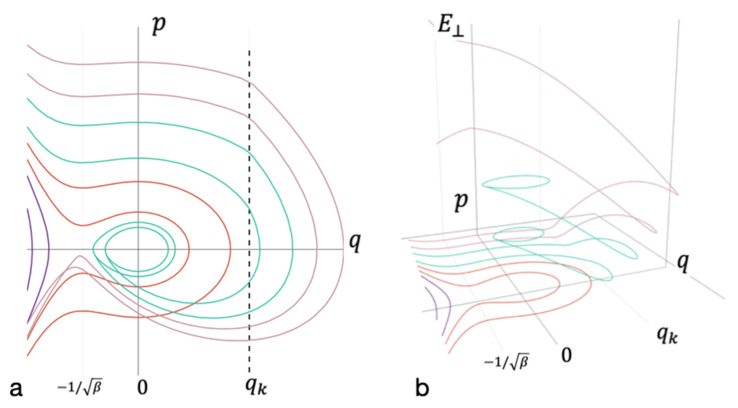
Phase diagram accounting for the energy redistribution between modes. (**a**) The projection of the phase space on the coordinate axis *q* and momentum *p* of oscillations of the entire cluster; (**b**) the axis of energy falling on the remaining degrees of freedom $E_{⊥}$. Trajectories that do not lead to bond formation are marked in purple. Red—trajectories that lead to the formation of short-lived bond without energy redistribution. Cyan—trajectories that lead to the formation of long-lived bonds. Magenta—trajectories that do not lead to the formation of long-lived bond after energy redistribution.

Energy redistribution between the degrees of freedom takes place at a sufficiently large nonlinear term in Equation (3). In the phase diagram, this area is separated by a vertical dashed line (see Appendix A and Figure 3). Rapid energy redistribution to other degrees of freedom begins when the trajectory crosses this line. Figure 3 shows that for a wide range of energies, energy dissipation is sufficient for the formation of a long-lived bond.

**Figure 4:**
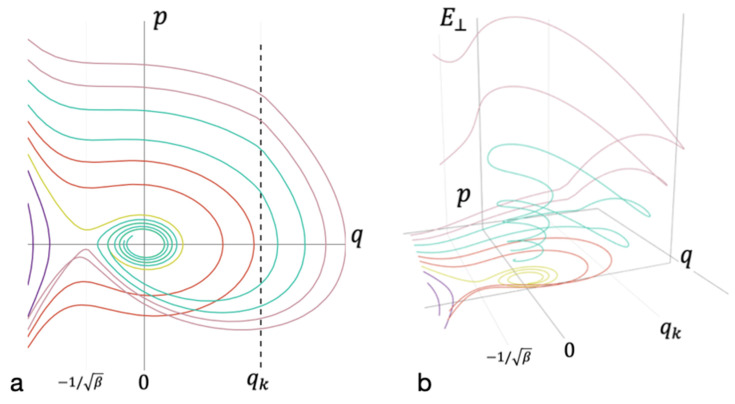
Phase diagram accounting for the dissipation and the energy redistribution between modes. (**a**) The projection of the phase space on the coordinate axis *q* and momentum *p* of oscillations of the entire cluster; (**b**) the axis of energy falling on the remaining degrees of freedom $E_{⊥}$. Trajectories that do not lead to bond formation are marked in purple. Yellow—trajectories that leaf to the formation of long-lived bond without energy redistribution. Red—trajectories that lead to the formation of short-lived bond without energy redistribution. Cyan—trajectories that lead to the formation of long-lived bonds. Magenta—trajectories that do not lead to the formation of long-lived bond after energy redistribution.

Figure 4 shows diagrams for the case with energy dissipation into the environment. The energy spectra of the system, in which the formation of long-lived bonds can take place, are very close to those shown in Figure 3. Therefore, the consideration begins with a simplified model, within which the dissipation to the external medium was assumed to be negligible (see Figure 3). Then, the consideration proceeded to the construction of phase portraits with nonzero dissipation (see Figure 4).

The methods used enabled us to reveal the stratification of phase flows in the system under consideration (see Equation (3)). Moreover, it was possible to determine separatrix lines (surfaces) and to find both threshold values: the potential energy of new bond formation, $E_{p}$, and the kinetic energy, $E_{k}$. When the latter is exceeded, the energy is redistributed from one degree of freedom to the others.

### 3.3. Von Neumann Entropy Approximation

The height of the kinetic barrier depends on the von Neumann entropy (see Equation (9)). The value of the von Neumann entropy significantly depends on the cluster configuration [55]. Computing the von Neumann entropy is generally a difficult task [64]. However, for some classes of graphs, the task is more manageable. Particularly, for fully connected graphs, it was possible to find the expression that approximated the von Neumann entropy. In this case, the von Neumann entropy is given by the asymptotic expression $H_{L}\approx \ln N$ [55,56].

**Figure 5:**
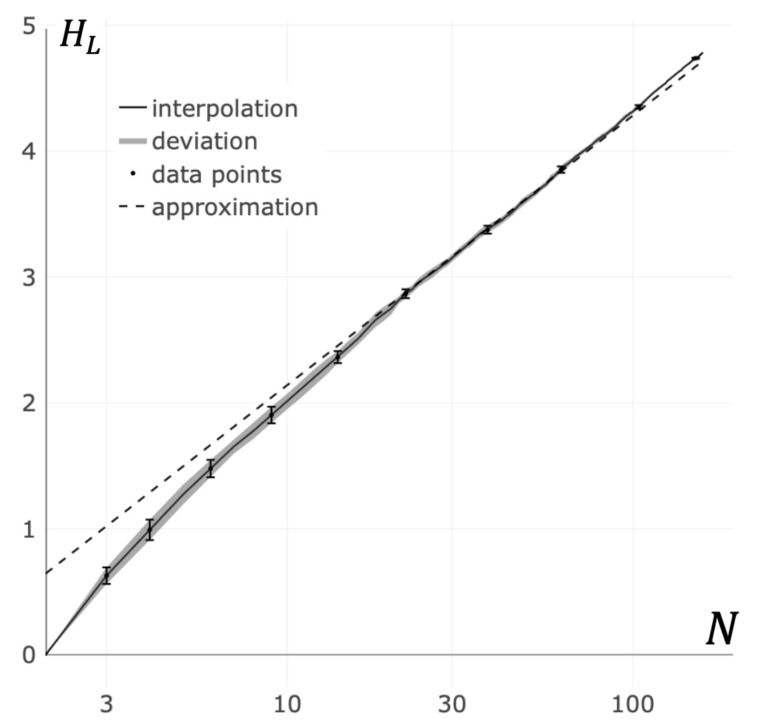
Dependence of the von Neumann entropy on the cluster size. The solid line is the interpolation of averaged entropy values for random bond graphs with constraints on the vertex degrees (see Appendix B). The area around solid line is the standard deviation in data. The dashed line is the approximation of the von Neumann entropy in accordance with Equation (10).

In real receptor clusters, the full connectivity of the corresponding graphs, as a rule, is not achieved due to steric constraints. In further consideration, the maximum number of bonds per receptor will be denoted as ξ. The results of our calculations of the von Neumann entropy for random receptor clusters are shown in Figure 5 (see Appendix B). It can be easily seen from Figure 5 that the von Neumann entropy for the random clusters with greater than 10 receptors (N>10) is approximated with high accuracy by the expression:

(10) $$H_{L}\approx \sigma \ln N$$

where σ is the coefficient of proportionality. From the results presented in Figure 5, it follows that for random graphs of bonds between receptors in a cluster, the best agreement between the data of numerical calculations and the data obtained from the Equation (10) is achieved at a value of σ=0.93.

### 3.4. Critical Size of Receptor Cluster

The value of the potential barrier $E_{p}$, as follows from Expression (8), does not depend on the total number of receptors in the cluster N. The value of the kinetic barrier $E_{k}$, as follows from Equations (9) and (10), hyperbolically depends on N: $E_{k}\;\propto \;1/N^{\left(1-\sigma \right)}$. Consequently, there is a certain $N_{*}$ at which $E_{k}=E_{p}$. Formally, when condition ($N>N_{*}$) is satisfied, the concentration of free receptors $C_{r0}$ on the cell surface (at which the rates of attachment and detachment of free receptors to clusters are the same) corresponds to the state of thermodynamic equilibrium. Within the framework of the developed approach, in real cells the condition $N\ll N_{*}$ is assumed to be fulfilled. Therefore, $E_{k}>E_{p}$.

For a nucleus smaller than the critical size, the rate of attachment of receptors to this nucleus is lower than the rate of detachment, and, on average, these smaller nuclei will degrade. When the critical size is exceeded, the attachment speed is greater than the detachment speed. Assuming the same cross-section for the attachment and detachment reactions, we obtain the following equation for the critical size (see Appendix C):

(11) $$N_{c}={\left(\frac{3\pi \varepsilon_{p}}{\varepsilon_{p}+\ln\left(C_{r}/C_{r0}\right)}\right)}^{\frac{1}{1-\sigma }}$$

where $\varepsilon_{p}=E_{p}/k_{B}T$ is the normalized bond breaking potential barrier, $C_{r}$ is the concentration of free receptors on the cell surface, and $C_{r0}$ is the saturated concentration of free receptors.

**Figure 6:**
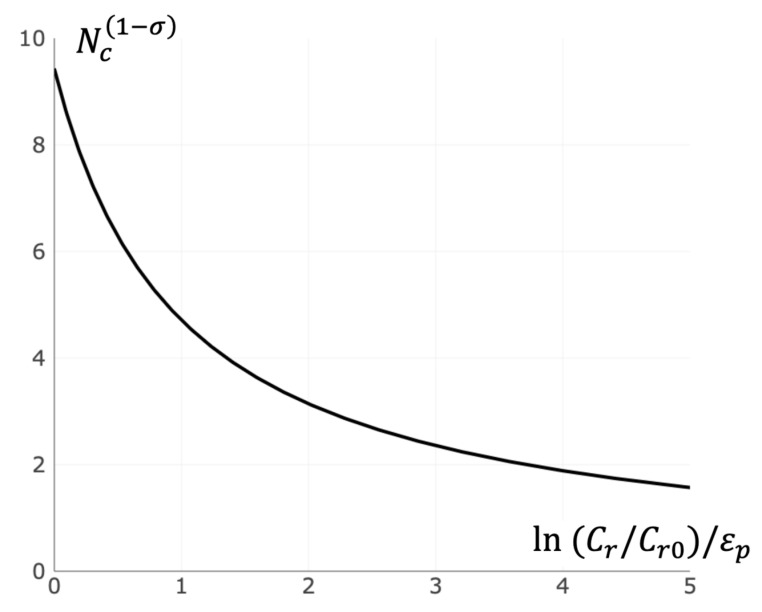
Dependence of the critical cluster size on the concentration of free receptors. The critical cluster size $N_{c}$ hyperbolically depends on logarithm of free receptor concentration $C_{r}$. Calculation was made in accordance with Equation (11).

The total number of receptors on the cell surface can be changed due to functional and pathological reasons, resulting in ligand sensitivity variation. In the developed approach, an increase in ligand sensitivity corresponds to a decrease of critical size $N_{c}$ due to an increase of $C_{r}$ (see Equation (11) and Figure 6). 

### 3.5. Heterogenous Nucleation Efficiency

Let us estimate the change in the formation rate of supercritical clusters upon the addition of ligands according to Equation (1). For this, by substituting Equation (7) into Equation (9), the expression for the heterogeneous nucleation efficiency (HNE) can be easily found:

(12) $$HNE\left(t\right)=\ln\frac{\dot{p_{l}}}{\dot{p_{0}}}\approx \frac{\xi N_{c}}{m_{r}\sigma \varepsilon_{p}}\sum_{l}m_{l}K_{l}C_{l}\left(t\right)$$

where $\dot{p_{l}}$ is the probability of forming a supercritical receptor cluster per unit time as a result of the interaction of receptors in the presence of ligands, and $\dot{p_{0}}$ is the relevant probability in the absence of ligands.

Using Equation (12), one could obtain the probability $p_{l}\left(t\right)$ of the ligand-induced formation of a supercritical cluster at time t:

(13) $$p_{l}\left(t\right)=1-\exp\left\{-\frac{1}{\tau_{r}}\int_{0}^{t}\exp\left[HNE\left(\tau \right)\right]d\tau \right\}$$

where $\tau_{r}=p_{0}/\dot{p_{0}}$ is the characteristic time of a supercritical cluster formation in the absence of ligands.

### 3.6. Homologous Series in T Cell Activation by Oligomeric MHC

**Figure 7:**
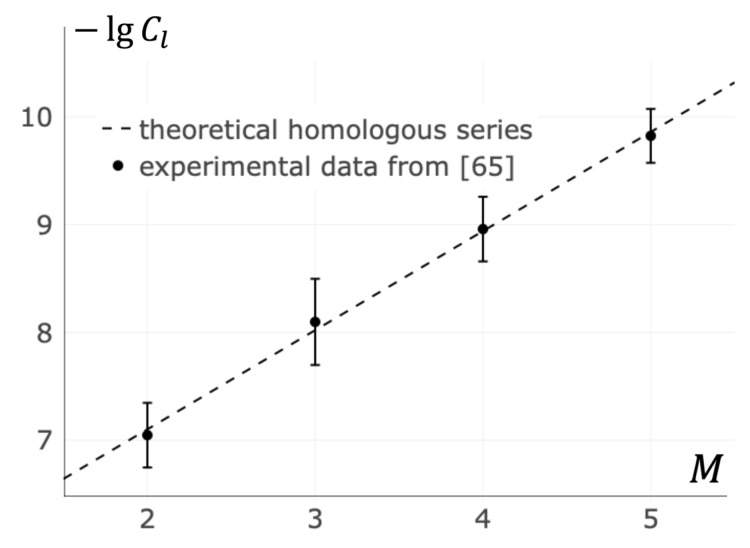
Dependence of T cell activation on oligomericity of the Major Histocompatibility Complex (MHC). The abscissa *M* shows the number of individual MHC in a synthetic MHC oligomer. The ordinate $-\mathrm{lg}C_{l}$ shows the minimal MHC oligomer concentration logarithm at which T cell activation occurs. The dashed line shows the “homologous series” predicted by the developed theory. The points with error bars represent the data of the experiments [65].

In light of the results received, novel methods for searching of the “homologous series” in several biologically important families of receptor-ligand systems become possible. Oligomeric ligands are of particular interest. In that case, receptor clusters nucleation depends only on ligands mass $m_{l}$ and the concentration of ligands $C_{l}\left(t\right)$. In particular, the homologous series for oligomers of Major Histocompatibility Complexes (MHC) was investigated (see Figure 7 and Appendix D). It was found that there is a linear dependence of concentration logarithm on oligomericity M:

(14) $$\ln C_{l}=\kappa -\theta \cdot M$$

where coefficients κ and θ are given by Equations (15) and (16):

(15) $$\kappa =\ln\left(-\frac{\tau_{r}}{t\gamma }\ln\left(1-p_{l}\left(t\right)\right)\right)$$

(16) $$\theta =\frac{\xi N_{c}K_{l}C_{\alpha }m_{m}}{m_{r}\sigma \varepsilon_{p}}$$

where $m_{m}$ is the single monomer mass and γ is the proportionality constant (see Appendix D).

Equation (14) is used to fit data from Cochran’s, Cameron’s, and Stern’s paper on the activation of T cells by Major Histocompatibility Complex oligomers [65]. The best agreement between Equation (14) and the data is achieved at κ=−5.26 and θ=0.92.

## 4. Discussion

The approach developed for the description of receptor cluster nucleation is a generalization of the classical theory of nucleation [2]. The focus is on the redistribution of energy over the degrees of freedom in the formation of new bonds between receptors. The effects of energy redistribution over the degrees of freedom made it possible to reveal the dependence of the critical cluster size $N_{c}$ on the concentration of free receptors present on the cell surface $C_{r}$ (see Equation (11)).

Energy redistribution over the degrees of freedom is an essentially dynamic effect and, as far as the authors know, has never been considered before in describing nucleation effects [66]. Our analysis of the dynamics of ligand–receptor complexes made it possible to establish how the rate of nucleation processes should depend on the mass of heterogeneous agents (ligands). We obtained an asymptotic expression for the kinetic barrier $E_{k}$ (see Equation (9)), exceeding which the effects of energy redistribution become decisive.

The attachment of ligands to receptors brings together the eigenfrequencies of oscillations in the cluster, thereby leading to an increase in the probability of the formation of long-lived bonds. With this in mind, we compared the rates of “heterogeneous” and “homogeneous” nucleation. The concentration of heterogeneous ligands $C_{l}$ exponentially enters the expression for the probability of formation of a supercritical receptor cluster twice (see Equations (12) and (13)). The approach developed seems to be quite general. It allows one to assess situations both with individual ligands and with a mixture of several types of ligands that competitively bind to receptors, including those with time-varying concentrations (see Equation (12)).

In the developed approach, the response of the cell to the appearance of specific ligands implies a particular sequence of events:

1. The formation of supercritical clusters by the mechanism of heterogeneous nucleation;
2. The growth of these clusters to a productive state that initiates cell’s intrinsic signaling pathways;
3. Signal transmission along the signaling pathway to the cell nucleus, where gene expression takes place.

Rigorously speaking, the expressions obtained in this work correspond to the first stage, which is assumed to be limiting. Indeed, the second stage seems to be inevitable from the point of view of general nucleation theory [3,4,5]. The third stage (intracellular signal transduction) is widely described in the literature as being extremely sensitive [35,36,37].

In the framework of Eyring’s classical theory [46], the attachment of one particle to another in the course of a chemical reaction is assumed to occur along one selected reaction coordinate, and the remaining (transverse) degrees of freedom serve only for energy relaxation. Generally, dynamics along several degrees of freedom can take on a more complex, substantially multidimensional character [67,68,69]. One example of such dynamics is the threshold energy redistribution within the cluster described above.

Traditionally, the thermodynamic approach has been used to determine the critical size of the nucleus [1,3,4,5]. In this case, the critical condition is determined by the equality of the chemical potentials in the supersaturated phase and the nucleus. However, within the thermodynamic approach, the energy equilibration after the formation of a bond is assumed to be fast enough. This work shows that, when this is not the case, the critical size of the nucleus and the height of the kinetic barrier of bond formation $E_{k}$ is determined by the dynamic parameters, particularly the masses of ligands and receptors.

To some degree, the developed approach is reminiscent to the “evolution of views” that took place in the description of epigenetic processes. In his pioneering work, Waddington proposed the concept of an epigenetic landscape, along which the state of the cell smoothly moves from one less “energetically favorable” to a more “energetically favorable” position [70]. After Rene Thom developed the theory of elementary catastrophes [71], he entered into correspondence with Waddington. They concluded that in the landscape, in principle, there can be “canopies”, as a result of which leaps should be present in the epigenetics of cells—elementary catastrophes [72]. The latter seems to be similar to the threshold effects in the redistribution of energy in the dynamics of clusters discussed in the present work.

From a formal mathematical point of view, the suggested approach for the description of the dissipation in an intermediate complex is isomorphic to the approach describing energy distribution processes in developed turbulent flows [73]. Within the framework of the turbulence theory, energy is fragmented during the decay of large vortices, and slow dissipation occurs mainly on the microscale [73,74,75]. The approach developed by us assumes fragmentation of energy by degrees of freedom and the slow dissipation of the fragmented energy in the vicinity of local equilibrium, which ultimately leads to the formation of a long-lived bond.

Due to the presence of an exponential in Equation (13), the summation over the types of ligands in Equation (12) is manifested in the effect of competitive ligands on the probability of cluster formation being multiplicative. That is, for a “weak” (nonspecific) ligand the HNE value is of the order of unity. So, the presence of the relevant term in Equation (12) will reduce the characteristic time of cluster nucleation by approximately e≈3 times. At the same time, the HNE value of the “strong” (specific) ligand can be on the order of 10. Therefore, the “strong” ligand by itself is capable of reducing the characteristic nucleation time by $e^{10}\approx 2\times 10^{4}$ times. In the presence of a “weak” ligand, the nucleation time decreases even more, decreasing by $e^{11}\approx 6\times 10^{4}$ times. This, in our opinion, can explain the observation of a significant increase in sensitivity to the specific ligands upon the addition of nonspecific ones [28].

The nature of the bonds between receptors in the cluster continues to be discussed [22]. One of the most promising areas of discussion concerns the clustering effects that are based on the attachment of receptors to the actin cytoskeleton located under the cell membrane [76]. In light of our consideration, actin filaments seem to be able to provide oscillations of receptors with a sufficiently high “quality factor”, which, in principle, facilitates intermodal energy transfer, making the developed approach of interest to specialists studying actin cytoskeleton dynamics [77].

Receptor clustering occurs in many types of cells [29,30,31,34]. In this regard, the developed approach should formally work when describing the processes of clustering receptors of various types of cells. From one side, Equation (13) seems to be rather general. From another side, the actual boundaries (limits) of its applicability require further research. At first glance, the developed approach seems to be oversimplified. Within its framework, many essential aspects of ligand–receptor interactions remain behind the scenes. However, considering the dynamic redistribution of energy over the degrees of freedom during the formation of critical receptor clusters makes it possible to consider the effects caused by the mass of interacting elements. In principle, this consideration makes it possible to assess the contribution of structurally similar (homologous) ligands to nucleation processes.

In particular, Equation (12) can be used for a qualitative comparison of similar ligands of the same origin (homologous). This is in analogy to the homologous series originally found by Vavilov N.I. for cereals [78]. Figure 7 shows the results of the calculations for the dependency of the rate of naive T cell receptor clustering on the MHC oligomericity. All of the ligands are represented by the points falling on a single line within the margin of error.

Equation (12) can be used to assess the sensitivity of platelets to conformational changes in the von Willebrand factor. This kind of ligand–receptor interaction is characterized by the “condensation” of receptor clusters on the linear structure of the von Willebrand factor [79,80]. Within the framework of the developed approach, this consideration is formally expressed in an increase of the effective local concentration of the ligands (monomers of the multimeric von Willebrand factor) at the platelet surface. Thus, von Willebrand factors, “heavier” in molecular weight, should make a noticeably greater contribution to platelet activation due to the clustering of GPIb receptors on their surface. Recently, similar experimental indications have been published [81,82].

## Data Availability

The data presented in this study can be reproduced by running the script available at https://doi.org/10.5281/zenodo.5525255 (accessed on 23 September 2021).

## Appendix A

In the problem under consideration, the equation of motion of receptors in a cluster has the following form:

(A1) $$M\ddot{X}=-kLX\left(I-\beta XL^{2}X\right)$$

where $X=diag\left(x_{1},y_{1},x_{2},y_{2}\ldots x_{N},y_{N}\right)$ is the matrix of receptor coordinates, $M=diag\left(M_{1},M_{1},M_{2},M_{2}\ldots M_{N},M_{N}\right)$ is the mass matrix, L is the Laplacian matrix for the graph of receptor bonds in the cluster, I is the identity matrix, k is the bond stiffness, and β is the nonlinear bond coefficient. The dimensions of the square matrices X, M, and L are given by the doubled number of receptors 2N×2N, where N is the number of receptors in the cluster.

The equation of motion of a sole receptor with one bond, if the rest are fixed, is as follows:

(A2) $$m\ddot{z}=-kz\left(1-\beta z^{2}\right)$$

where m is the mass of one receptor, and z is the displacement along the bond axis.

We will assume that the bond breaks at an unstable equilibrium point, defined by $1-\beta z_{m}^{2}=0$ (see Equation (A2)). We obtain Expression (A3) for the distance at which the bond breaks and, integrating the right-hand side of Equation (A2), we obtain an expression for the bond energy (A4):

(A3) $$z_{m}=1/\sqrt{\beta }$$

(A4) $$E_{p}=\overset{z_{m}}{\underset{0}{\int }}kz\left(1-\beta z^{2}\right)dz=\frac{k}{4\beta }$$

Let us carry out the spectral decomposition of Equation (A1) and write out the ith component corresponding to one oscillation mode:

(A5) $$m\mu_{i}{\ddot{q}}_{i}=-k\lambda_{i}q_{i}\left(1-\beta \lambda_{i}^{2}q_{i}^{2}\right)$$

where $\mu_{i}$ is the dimensionless mass of the oscillation mode, $q_{i}$ is the phase coordinate of the oscillation mode, and $\lambda_{i}$ are eigenvalues of matrix L.

The characteristic order of frequencies is given by Equation (A6), and the specific frequency of the mode is given by Equation (A7):

(A6) $$\omega_{0}=\sqrt{k/m}$$

(A7) $$\omega_{i}=\omega_{0}\sqrt{\lambda_{i}/\mu_{i}}$$

When applied to a system with multiple oscillation modes, the quasi-periodicity condition has the form Δω/f>1, where Δω is the average distance between the excited and the neighboring modes, and f is the separatrix size [57]. Concerning our system, the condition for the destruction of quasi-periodicity can be estimated as:

(A8) $$\beta \lambda_{i}^{2}q_{im}^{2}>3\pi \omega_{i}^{2}/2N\omega_{0}^{2}=3\pi \lambda_{i}/2N\mu_{i}$$

where $q_{im}$ is the maximum value of the phase coordinate of the mode, and N is the number of receptors in the cluster.

The following expression is the energy for each mode:

(A9) $$E_{i}=m\mu_{i}{q^{\prime }}_{im}^{2}/2=m\mu_{i}\omega_{i}^{2}q_{im}^{2}/2=k\lambda_{i}q_{im}^{2}$$

Then the total energy of oscillations will be equal to:

(A10) $$E=\overset{2N}{\underset{i=1}{\sum }}E_{i}=k\overset{2N}{\underset{i=1}{\sum }}\lambda_{i}q_{im}^{2}\approx kq_{m}^{2}\overset{2N}{\underset{i=1}{\sum }}\lambda_{i}$$

where $q_{m}$ is the RMS value of the phase coordinate.

Substituting Equation (A10) into Equation (A8) and taking the logarithm, we get the following:

(A11) $$\varphi_{i}=\ln\frac{4\beta NE}{3\pi k}+\ln\frac{\lambda_{i}}{\sum_{j}\lambda_{j}}+\ln\mu_{i}>0$$

where $\varphi_{i}$ is the dimensionless characteristic of the mode stochastization.

To assess the destruction of the quasi-periodicity of motion in all modes, we introduce the dimensionless characteristic of the system stochastization:

(A12) $$\Phi =\overset{2N}{\underset{i=1}{\sum }}\frac{E_{i}\varphi_{i}}{E}=\ln\frac{4\beta NE}{3\pi k}+\overset{2N}{\underset{i=1}{\sum }}\frac{\lambda_{i}}{\sum_{j}\lambda_{j}}\left(\ln\frac{\lambda_{i}}{\sum_{j}\lambda_{j}}+\ln\mu_{i}\right)$$

For a more convenient representation, we introduce two quantities: the von Neumann entropy of the Laplacian matrix of the graph of connections $H_{L}$ (see Equation (A13)) и and the mass cross-entropy Λ (see Equation (A14)).

(A13) $$H_{L}=-\overset{2N}{\underset{i=1}{\sum }}\frac{\lambda_{i}}{\sum_{j}\lambda_{j}}\ln\frac{\lambda_{i}}{\sum_{j}\lambda_{j}}>0$$

(A14) $$\Lambda =\overset{2N}{\underset{i=1}{\sum }}\frac{\lambda_{i}}{\sum_{j}\lambda_{j}}\ln\mu_{i}\geq 0$$

The boundary quasi-periodicity energy $E_{q}$ will be reached at Φ=0 and can be expressed as the following:

(A15) $$E_{k}=\frac{k}{4\beta }\frac{3\pi }{N}\exp\left(H_{L}-\Lambda \right)=3\pi E_{p}\exp\left(H_{L}-\Lambda \right)/N$$

Suppose we average all possible rearrangements of ligands over receptors in the cluster. In that case, the dimensionless masses of the oscillation modes $\mu_{i}$ become equal to each other, as well as the ratio of the mass of the cluster with ligands to the mass of the receptors of this cluster μ:

(A16) $$\mu =1+\overset{2N}{\underset{i=1}{\sum }}\frac{m_{i}}{N\cdot m}=1+\varkappa$$

where $m_{i}$ is the ith ligand mass, and ϰ is the ligands mass ratio to the mass of the receptors in the cluster.

Then a simplified expression can be obtained for $E_{k}$:

(A17) $$E_{k}=3\pi E_{p}\exp H_{L}/\left(1+\varkappa \right)N$$

where $m_{c}$ is the total mass of the cluster.

## Appendix B

We seek approximation for von Neumann entropy of Laplacian matrix $H_{L}$ in the form of linear dependence on cluster size N logarithm:

(A18) $$H_{L}\approx \sigma \ln N$$

where σ is the coefficient of proportionality.

To obtain the data needed to fit the proportionality coefficient, we generated random graphs and computed their von Neumann entropies (see Equation (4)). We restricted the maximum number of receptor bonds ξ by generating random graphs with limited vertex degrees [54]. After generating 100,000 graphs with different ξ, they were grouped by the number of vertices N. These dependencies were fitted against Equation (A18), and at a number of receptors greater than 10 (N>10) were approximated with high accuracy. There was practically no difference between ξ=3 and ξ=7. The best agreement between Equation (A18) and the data is at σ=0.93.

## Appendix C

For small sizes of receptor clusters, shedding receptors is faster than attaching new receptors. At larger sizes of receptor clusters, attaching may become faster than shedding. If it exists, the size at which the speeds are equal is called a critical size [3,4,5,6].

To determine this size, we found the dependence of shedding and attaching speeds. Firstly, the speed of shedding receptors from cluster $v_{-}\left(N\right)$ is determined by reaction cross-section and potential barrier (see Equation (1)):

(A19) $$v_{-}\left(N\right)=k_{-}s_{-}\left(N\right)\exp\left(-E_{p}/k_{B}T\right)$$

where $k_{-}$ is the speed of thresholdless reaction, $s_{-}\left(N\right)$ is the shedding reaction cross-section, $E_{p}$ is the energy of potential barrier, $k_{B}$ is the Boltzmann constant, and T is the temperature.

Similarly, we obtain the expression for the speed of the attachment of new receptors to sufficiently large clusters ($N>N_{*}$ see Section 3.4) speed $v_{+}^{*}\left(N,C_{r}\right)$:

(A20) $$v_{+}^{*}\left(N,C_{r}\right)=C_{r}k_{+}s_{+}\left(N\right)\exp\left(-\left(E_{p}-E_{f}\right)/k_{B}T\right)$$

where $C_{r}$ is the concentration of free receptors around the cluster, $k_{+}$ is the speed of the thresholdless reaction, $s_{+}\left(N\right)$ is the attaching reaction cross-section, and $E_{f}$ is the energy of free receptors.

For smaller clusters ($N<N_{*}$), the kinetic barrier is larger than the potential barrier and therefore the expression for the speed of the attachment of new receptors speed $v_{+}\left(N,C_{r}\right)$ takes form:

(A21) $$v_{+}\left(N,C_{r}\right)=C_{r}k_{+}s_{+}\left(N\right)\exp\left(-\left(E_{k}\left(N\right)-E_{f}\right)/k_{B}T\right)$$

where $E_{k}\left(N\right)$ is the energy of the kinetic barrier.

Let us define the saturated concentration $C_{r0}$ of free receptors as one that satisfies this property:

(A22) $$v_{+}^{*}\left(N_{*},C_{r0}\right)=v_{-}\left(N_{*}\right)$$

We substitute Equations (A19) and (A20) into Equation (A22) to find expression for saturated concentration. An approximate expression is derived by assuming that cross-sections of shedding and attaching are equal, as follows:

(A23) $$C_{r0}=\frac{k_{-}s_{-}\left(N_{*}\right)}{k_{+}s_{+}\left(N_{*}\right)}\exp\left(-\frac{E_{f}}{k_{B}T}\right)\approx \frac{k_{-}}{k_{+}}\exp\left(-\frac{E_{f}}{k_{B}T}\right)$$

Let us define the critical size of the cluster $N_{c}$ at the concentration of free receptors $C_{r}$ as one that satisfies this property:

(A24) $$v_{+}\left(N_{c},C_{r}\right)=v_{-}\left(N_{c}\right)$$

We substitute Equations (A19) and (A21) into Equation (A24) to find another expression for saturated concentration.

(A25) $$C_{r}\exp\left(-\frac{E_{k}\left(N\right)-E_{p}}{k_{B}T}\right)=\frac{k_{-}s_{-}\left(N_{*}\right)}{k_{+}s_{+}\left(N_{*}\right)}\exp\left(-\frac{E_{f}}{k_{B}T}\right)=C_{r0}$$

We proceed with finding the expression for the energy of the kinetic barrier:

(A26) $$E_{k}\left(N_{c}\right)=E_{p}+k_{B}T\ln\left(C_{r}/C_{r0}\right)$$

Another expression for the energy of kinetic barrier can be found by substituting Approximation (10) into Equation (9):

(A27) $$E_{k}\left(N\right)=\frac{3\pi }{N^{1-\sigma }}E_{p}$$

where σ is the coefficient of proportionality from the approximation.

Finally, by combining Equations (A26) and (A27), we obtain the expression for critical cluster

(A28) $$N_{c}={\left(\frac{3\pi \varepsilon_{p}}{\varepsilon_{p}+\ln\left(C_{r}/C_{r0}\right)}\right)}^{\frac{1}{1-\sigma }}$$

where $\varepsilon_{p}=E_{p}/k_{B}T$ is the normalized bond-breaking potential barrier.

## Appendix D

Equations (13) and (12) should formally describe receptor cluster nucleation in various types of cells. In practice, the direct comparison of different cell types is hindered by variation in the critical cluster size $N_{c}$ (see Results Section 3.4). On the contrary, the influence of characteristics of ligands on cell response does not have this problem since the critical cluster size stays constant. Oligomeric ligands are of particular interest. In that case, receptor clusters’ nucleation depends only on ligands mass $m_{l}$ and the concentration of ligands $C_{l}\left(t\right)$.

The local concentration of ligand-binding sites increases drastically when the oligomeric ligand is near the cell surface. This large concentration is assumed to be similar between oligomers of different sizes because ligand monomers are at the same distance. Therefore, the local concentration of ligands is assumed to be some constant $C_{\alpha }$ when the oligomer is near the cell surface and zero otherwise. The proportion of the time that oligomers spend near the cell’s surface is proportional to oligomer’s concentration in solution $C_{l}$:

(A29) $$t_{v}/t=\gamma C_{l}$$

where $t_{v}$ is the time oligomers spent near the cell surface, t is the overall time, and γ is the proportionality constant.

After substituting Equation (A29) into Equations (12) and (13) we obtain the expression for the probability of receptor cluster nucleation $p_{l}\left(t\right)$:

(A30) $$p_{l}\left(t\right)=1-\exp\left(-\frac{t\gamma C_{l}}{\tau_{r}}\exp\left(\frac{\xi N_{c}K_{l}C_{\alpha }m_{m}}{m_{r}\sigma \varepsilon_{p}}M\right)\right)$$

where $\tau_{r}$ is the characteristic time of a supercritical cluster formation in the absence of ligands, ξ is the maximum number of bonds per receptor, $N_{c}$ is the critical size of receptor cluster, $m_{m}$ is the mass of one ligand monomer, $m_{r}$ is the receptor mass, σ is the coefficient of proportionality for von Neumann entropy approximation, $\varepsilon_{p}$ is the normalized bond-breaking potential barrier, and M is the number of monomers in an oligomer (oligomericity).

After a series of transformations on Equation (A30), we rewrite it as a linear dependence of the concentration logarithm on oligomericity:

(A31) $$\ln C_{l}=\kappa -\theta \cdot M$$

where coefficients are given by Equations (A32) and (A33):

(A32) $$\kappa =\ln\left(-\frac{\tau_{r}}{t\gamma }\ln\left(1-p_{l}\left(t\right)\right)\right)$$

(A33) $$\theta =\frac{\xi N_{c}K_{l}C_{\alpha }m_{m}}{m_{r}\sigma \varepsilon_{p}}$$

Equation (A31) is used to fit data from Cochran’s, Cameron’s, and Stern’s paper on the activation of T-cells by Major Histocompatibility Complex oligomers [65]. The best agreement between Equation (A31) and the data is at κ=−5.26 and θ=0.92.
